# Recent Advances Toward the Discovery of Drug-Like Peptides *De novo*

**DOI:** 10.3389/fchem.2015.00069

**Published:** 2015-12-18

**Authors:** Michael Goldflam, Christopher G. Ullman

**Affiliations:** ^1^Isogenica Ltd.Little Chesterford, UK; ^2^Paratopix Ltd.Bishop's Stortford, UK

**Keywords:** directed evolution, display systems, peptide drug discovery, peptides and derivatives, stability, non-natural amino acids, cyclic peptides

## Abstract

Peptides are important natural molecules that possess functions as diverse as antibiotics, toxins, venoms and hormones, for example. However, whilst these peptides have useful properties, there are many targets and pathways that are not addressed through the activities of natural peptidic compounds. In these circumstances, directed evolution techniques, such as phage display, have been developed to sample the diverse chemical and structural repertoire of small peptides for useful means. In this review, we consider recent concepts that relate peptide structure to drug-like attributes and how these are incorporated within display technologies to deliver peptides *de novo* with valuable pharmaceutical properties.

Peptides have traditionally been considered a poor choice for drug candidates owing to their low bioavailability, susceptibility to proteolysis, and rapid clearance (Craik et al., 2013; Otvos and Wade, 2014). Nevertheless, peptides are powerful natural mediators of biological processes (Kastin, 2006) and over 60 peptide products have been approved by the FDA with global sales estimated at US$14.7b in 2011 (seven of which have been generating sales in excess of US$0.5b) with a further 140 in clinical development (Reichart, 2010; Hamzeh-Mivehroud et al., 2013; Kaspar and Reichert, 2013). As a drug modality, peptides, and peptidomimetics occupy the interface between small molecule and larger biological drugs with the potential to have the specificity of antibodies and the bioavailability of small molecules. Yet, although this prospect has yet to be fully realized, there has been significant recent progress in this direction (Craik et al., 2013; Otvos and Wade, 2014). Peptides provide the researcher with interesting options for discovery, optimization, and manufacture as they can be synthesized chemically or expressed by recombinant systems. For example, recombinant methods of discovery, such as directed evolution display systems, have isolated novel sequences in addition to those found in nature. Additionally, chemical methods of improvement can be employed to optimize stability, affinity, or bioavailability. In this short review, we will briefly describe biological methods for discovering new peptide entities and the chemical methods that are implemented to improve pharmacological properties.

Early combinatorial peptide synthesis methods described synthesis of hexamer peptide libraries on pins or beads, but chemical synthesis methods have since improved significantly in scale and coupling efficiencies (Geysen and Mason, 1993; Lam et al., 1993; Breitling et al., 2009). However, recombinant display systems are capable of screening libraries that are orders of magnitude larger. These display methods are based upon the principle that each peptide maintains a link between itself and the nucleic acid code from which it was expressed so that it can be identified ultimately from a diverse population by nucleic acid sequencing, following cycles of binding, washing, and enrichment (Figure 1A). This genotype-phenotype link is exhibited by displaying the peptide with its genetic material encapsulated in a virus particle (e.g., phage display), or on the surface of a cell which contains the genetic information (e.g., yeast or bacterial display) or directly linked to the nucleic acid through non-covalent or covalent means (e.g., CIS display, mRNA display, and ribosome display) (Figure 1B; Ullman et al., 2011).

**Figure 1:**
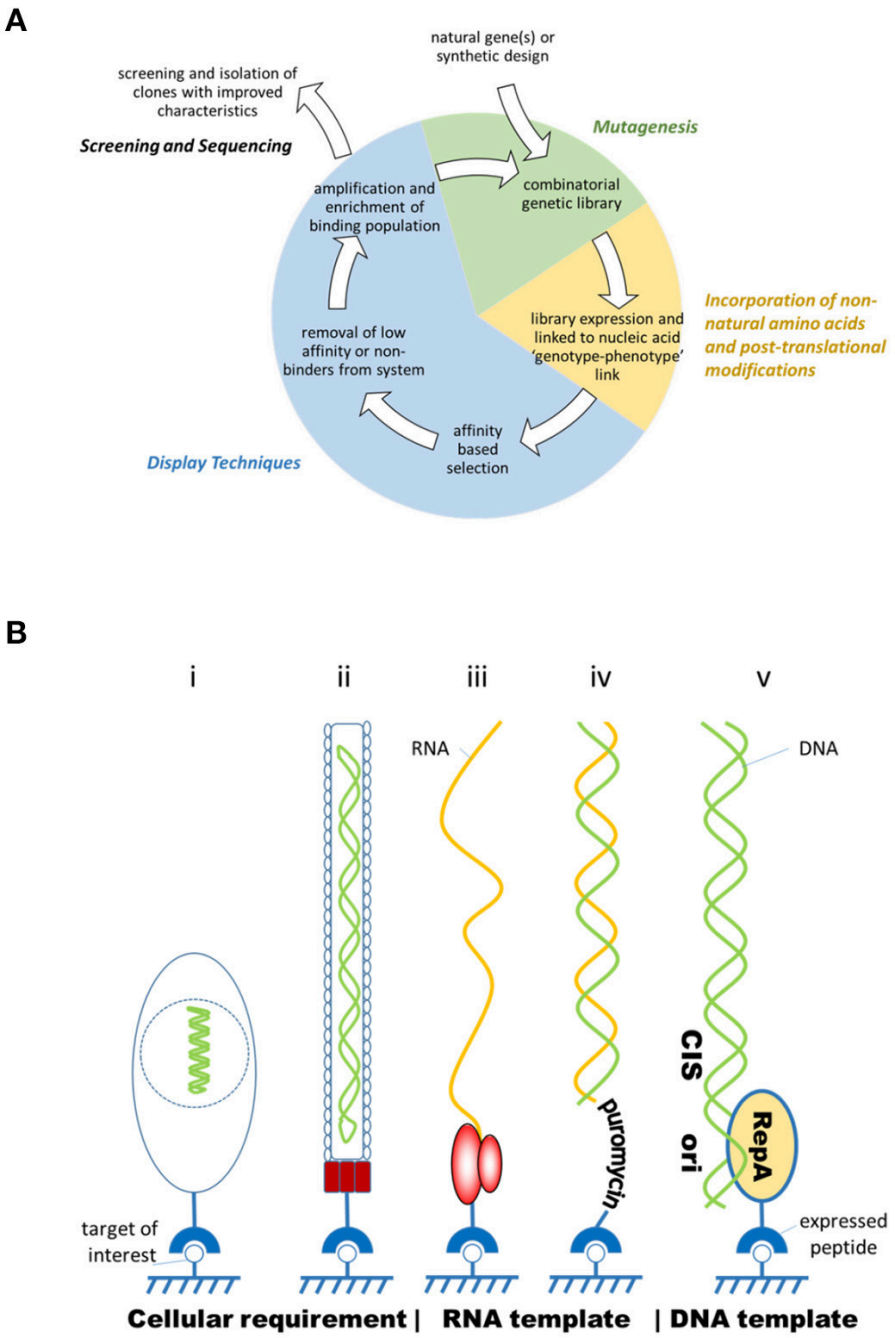
**(A)** Overview of the process of directed evolution. Combinatorial genetic libraries are created through mutagenesis, then transcribed and translated into peptides which retain a physical link with their encoding genetic information. Application of selection pressure, such as cycles of washing, amplification and further selection, enrich the binding clones within the population. Ultimately, binding peptides are identified through sequencing of the genetic material. **(B)** Diagram showing different formats of display where the peptide is expressed on the surface of a yeast or bacterium (i) (yeast/bacterial display); or a bacteriophage (ii) (phage display); or in complex with a ribosome (iii) (ribosome display); linked to RNA through puromycin (iv) (mRNA display); or bound to DNA through the *cis* activity of a DNA binding protein (v) (CIS display). In all these examples the peptide is associated with its coding sequence maintaining a genotype-phenotype link and is free to associate to its target (adapted from Ullman et al., 2011).

Cellular display approaches require cells to be transformed, a process that delivers the coding sequence of the peptide into a host cell for expression and display. In phage display, filamentous or T7 lytic bacteriophages are produced with the peptide expressed in fusion with a viral coat protein (usually pIII or pVIII in the case of M13). Within a diverse library, each phage particle carries a different peptide clone. This library is “panned” to select binding peptides amongst the population, panning conditions being balanced so that stringency is sufficient to isolate rare high affinity peptides from a background of poor or non-binding members (Smith and Petrenko, 1997; Sidhu et al., 2000; Hoogenboom, 2005; Levin and Weiss, 2006; Sergeeva et al., 2006; Krumpe and Mori, 2007; Hamzeh-Mivehroud et al., 2013). Molecules isolated by phage display are now just reaching the market, for example Peginesatide, a novel PEGylated peptide. Peginesatide acts as an agonist of the erythropoietin receptor and was approved by the FDA for the treatment of anemia due to chronic kidney disease in adult patients on dialysis (Wrighton et al., 1996; Macdougall et al., 2009; Reichart, 2010). Although effective, Peginesatide has now been withdrawn due to safety concerns. Phage display has been used successfully for *in vivo* panning (“biopanning”) in animal and human subjects to isolate tissue-specific peptides and those that can transgress biological barriers such as the skin, blood brain barrier, intestinal tract, and cell membrane (Arap et al., 2002; Frenkel and Solomon, 2002; Gao et al., 2002; Duerr et al., 2004; Chen et al., 2006; Sergeeva et al., 2006; Giordano et al., 2010). Yeast and bacterial display are technically related methods where peptides or proteins can be expressed on the surface of cells in fusion with Aga2p (yeast) or bacterial flagellin, outer membrane proteins, such as OmpA, or albumin binding protein and XM' sequence of staphylococcal protein A followed by affinity based cell-sorting methods (Daugherty, 2007; Gai and Wittrup, 2007; Rockberg et al., 2008).

Cell-free methods of peptide display avoid the need for transformation and therefore are not bound by the practical limitations of this procedure [typically up to 10^10^ clones for phage although 10^12^ has been reported (Sidhu et al., 2000)], but can reach a theoretical diversity of 10^14^ clones. Therefore, cell-free methods potentially allow greater coverage of the sequence space. These methods use the transcription and translation machinery extracted from prokaryotic or eukaryotic cell lysates. In ribosome display, ribosomes are stalled on the mRNA template through the absence of a stop codon and the nascent peptide remains in a complex with the ribosomes; a C-terminal peptide spacer facilitates correct folding of the displayed polypeptide free from steric hindrance by the ribosomal tunnel (Mattheakis et al., 1994; Hanes and Plückthun, 1997; Douthwaite and Jackson, 2012). A related technique, mRNA display, differentiates itself from ribosome display by the formation of a covalent link between the template and the expressed peptide via puromycin. Puromycin is carried on a DNA primer appended to the mRNA template and mimics amino-acyl tRNA, binding covalently to the nascent peptide as a result of the peptidyl transferase activity of the ribosome (Nemoto et al., 1997; Roberts and Szostak, 1997; Douthwaite and Jackson, 2012). The RNA based systems can be very sensitive to RNAse degradation and reconstituted transcription translation systems have been employed to reduce this effect, for example the PURE system (Shimizu et al., 2001, 2005). DNA based cell-free systems such as, CIS display, which harnesses the ability of a DNA-binding protein (RepA) that exclusively binds back to its encoding template, offer the advantage of speed, ease of use, and template stability over RNA (Odegrip et al., 2004; Eldridge et al., 2009; Patel et al., 2013).

These aforementioned display systems enable vast libraries to be screened based upon natural L-amino acids. However, in order to drive efficacy, non-natural additions may be required. In this respect we can gain insight from natural solutions. An exemplar of an effective natural peptide drug is the macrocycle cyclosporin A (CsA; Figure 2A). Originally isolated from the fungus *Tolypocladium inflatum* it has been intensively studied to understand the correlation between structural features and pharmaceutically relevant properties (Loosli et al., 1985; Kessler et al., 1990; Ko and Dalvit, 1992; Jin and Harrison, 2002). These studies have revealed four key features responsible for CsA's cell permeability, serum stability, and oral bioavailability: a cyclic backbone; incorporation of seven N-methyl groups; four intramolecular hydrogen bond donors; incorporation of three non-natural amino acid. Thus, CsA exhibits exquisite drug-like properties despite violating medicinal chemistry's traditional rule of 5: No more than 5 hydrogen bond donors and 10 acceptors, a molecular mass of less than 500 Da, and a logP coefficient no higher than 5 (Lipinski et al., 2001).

**Figure 2:**
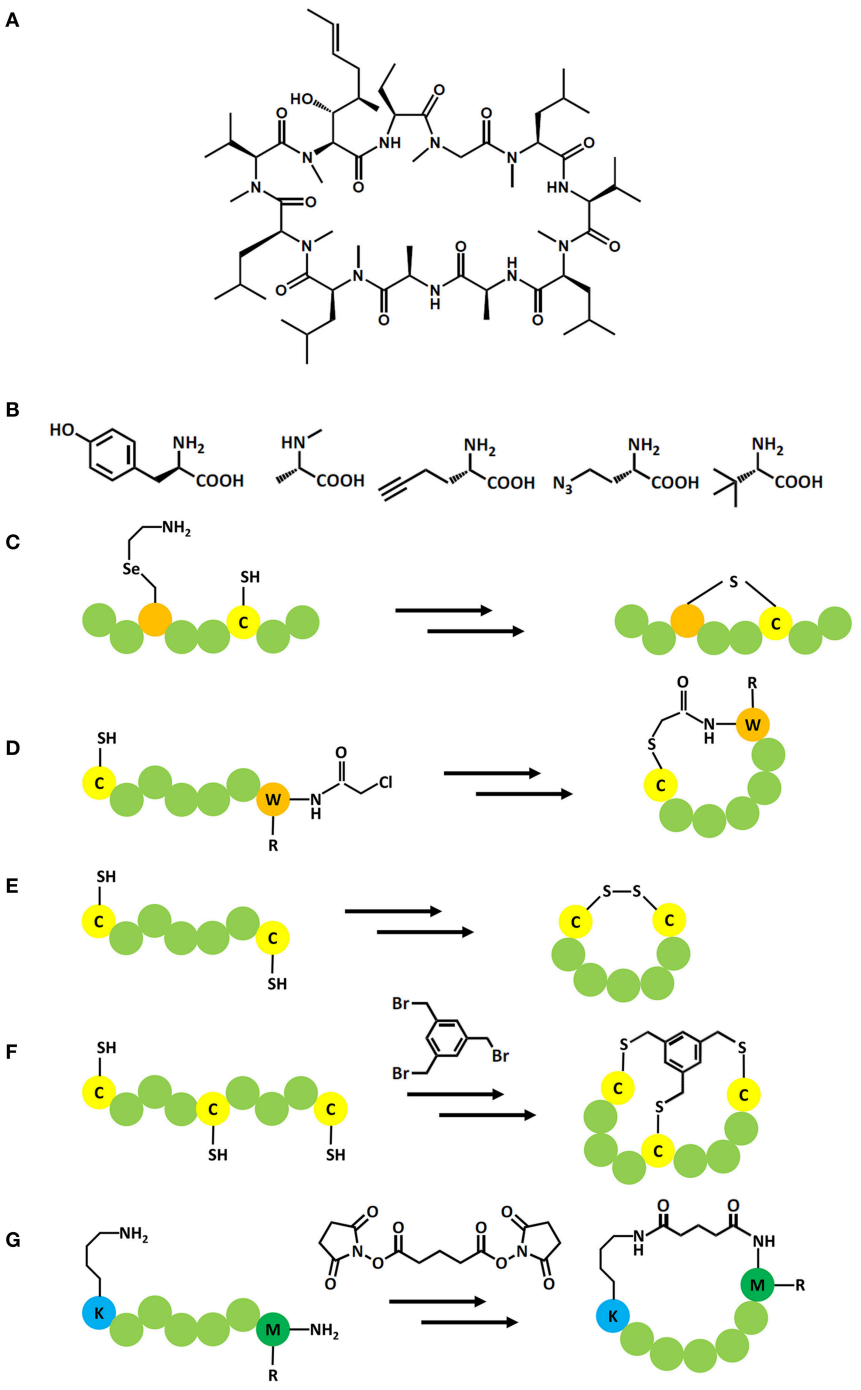
**(A)** Structure of CsA; **(B–D)** Incorporation of non-natural amino acids in peptide display systems: **(B)** Examples for the wide scope of non-natural amino acids that can be included in display systems e.g., from left to right, D-tyrosine, N-Methyl-L-alanine, (S)-2-amino-4-azidobutanoic acid, (S)-2-aminohex-5-ynoic acid, (S)-2-amino-3,3-dimethylbutanoic acid; **(C)** Incorporation of 4-selenalysine allows the formation of a lantipeptide with a cysteine residue; **(D)** Incorporation of amino acids with a chloroacetylated N terminus allows the formation of thioether bond with a downstream cysteine. **(E–G)**: Posttranslational modifications in peptide display systems: **(E)** Formation of a cyclic peptide via a disulphide bond; **(F)** Formation of a cyclic peptide by cross linking two or three cysteine residues with activated aryls, e.g., tris-(bromomethyl)benzene; **(G)** Formation of a cyclic peptide by cross linking the N terminus with a downstream lysine via disuccinimidyl glutarate.

Investigations into other natural products and synthetic peptide libraries have further highlighted the importance of cyclisation and N-methylation for pharmacological properties to the extent that these attributes reduce flexibility and favor resistance to proteolysis, and provide a capacity to permeate biological membranes through a reduction of the number of amide hydrogen bond donors through N-methylation (Biron et al., 2008; White et al., 2011). In addition, both cyclisation and N-methylation support the formation of intramolecular hydrogen bonds which further decreases flexibility and solvation (Biron et al., 2008; White et al., 2011). Interestingly, most studies of passive membrane perfusion by cyclic peptide libraries have demonstrated that the impact of N-methylation on membrane permeability was highly position dependent and not necessary correlated with increasing N-methyl content (Biron et al., 2008; White et al., 2011). Instead, optimal N-methylation patterns favored conformations with reduced hydrogen bonding potential for solvation by water, therefore reducing the desolvation of the peptide before entering the membrane while simultaneously concealing the hydrophilic backbone from the membrane environment. Furthermore, it was established for CsA that passive transcellular absorption might be a general characteristic of a macrocycle that can assume multiple interconverting conformations between the aqueous environment and the lipophilic environment of the membrane (Bockus et al., 2013; Wang et al., 2015). Finally, it is generally accepted that flexible molecules face a higher entropic barrier upon binding to their target than restrained molecules and often have lower affinity than cyclic counterparts. The same effect might influence the translocation of molecules from the highly mobile aqueous phase to the reduced entropic environment of the membrane (Bockus et al., 2013). Indeed, Veber et al. have shown that the number of rotatable bonds is a better predictor of bioavailability than molecular weight. These findings have helped to drive new thinking and interest in designing novel molecules that occupy the chemical space beyond the borders dictated by the Lipinski rules of 5 (Veber et al., 2002). For macrocyles a recent study highlighted an increase of clinical candidates administered by oral routes (43%) compared to already registered drugs (28%). In the subgroup of cyclic peptides CsA is currently the only oral prescribed drug. However, 3 out of 11 cyclic peptides in trials have biophysical properties similar to CsA and are tested for oral applications (Giordanetto and Kihlberg, 2014).

Non-standard natural product peptides have provided excellent leads for drug discovery in the field of antibiotics. However, most of these peptides are synthesized by non-ribosomal peptide synthetases, as is CsA. Although these modifications can be synthesized chemically, the capacity of molecular evolution methods in biological systems to generate libraries of trillions of peptides is extremely attractive (Ullman et al., 2011). Approaches to reengineer the complex gene cluster involved in the synthesis of such peptides has proved to be very challenging, however, several modifications have been made to molecular display technologies to produce peptides incorporating such features. These technologies can be broadly classified into methodologies that are based on the incorporation of non-natural amino acids and those that are based on post-translational modification of encoded peptides.

## Improving pharmacological properties of peptides by post-translational modification

The first post-translational modification of a genetically encoded phage peptide library involved cyclisation through disulphide-bridge formation in which two invariant cysteine residues flanked a random amino acid sequence (Figure 2E). Such cyclic peptides had between 100 and 1000 fold higher affinities than linear peptides isolated for the same target (O'Neil et al., 1992; McLafferty et al., 1993), yet the sensitivity of the bond to reducing environments encouraged the development of chemistry-based cyclisation methods to obtain redox insensitive macrocyles. For example, a modification of mRNA display based on disuccinimidyl glutarate, which cross-linked the N-terminus and an internal lysine sidechain (Figure 2G) to identify a cyclic peptide from a trillion member library that bound to Gα_i1_ with high affinity (K_*i*_ = 2 nM; Millward et al., 2005). The cyclic peptide showed 15-fold improved affinity and 2.5-fold improved proteolytic stability compared to the linear motif (Millward et al., 2007).

The concept of using organic chemistry based cyclisation methods to improve the properties of DNA encoded peptides was further advanced by installing an activated aryl (tris-(bromomethyl)benzene) to crosslink phage-displayed peptide libraries containing three invariant cysteines into bi-cyclic peptides (termed “bicycle”; Figure 2F; Heinis et al., 2009). Selection with phage display against human plasma kallikrein yielded specific inhibitors in the mid nM range which could be further improved by affinity maturation to IC_50_ 1.7 nM. The linear analogs did not show any inhibition at up to 10 μM, thereby underlining the importance of the cyclic structure of the peptide. The high specificity for human over mouse kallikrein and other proteases was reasoned to be a result of the large interaction interface between bicyclic peptide and target, a potential advantage over mono-cyclic peptides (Heinis et al., 2009). Activated aryls have been also used in the selection of monocyclic peptides expressed by mRNA display (Schlippe et al., 2012).

Post-translational modifications to improve pharmacological properties of displayed peptides can also be introduced enzymatically. Several approaches have been described in the literature, e.g., the introduction of azoline moieties in peptide backbones (Goto et al., 2014) or head-to-tail cyclisation via an split-intein approach within cells using phenotype-based selection rather than display. Kritzer et al. used this approach to select candidates from a cyclic octamer peptide library, which were able to rescue a yeast model of α-synuclein toxicity and able to prevent neuron loss in a *C. elegans* model. Although the use of a yeast platform limits the available library size, it allows selection based on phenotype which has the advantage that candidates are selected for efficacy rather than affinity and toxic sequences are avoided (Kritzer et al., 2009). Yeast have also been reprogrammed for the incorporation of non-natural amino acids through orthologous amino acyl tRNA synthetase/tRNA pairs (Chin, 2011). Recently, sidechain-to-tail cyclization through ligation of 1,3- or 1,2-aminothiol functionalities within non-natural amino acids and the C-terminus has been reported enabling the isolation of improved streptavidin binding peptides from a 144-member library produced inside cells (Frost et al., 2015).

## Improving pharmacological properties of peptides by incorporation of non-natural amino acids

Two major routes have been explored to express peptides with non-natural amino acids: Firstly, exploiting the ability of native aminoacyl-tRNA synthetases to mischarge tRNAs, and secondly performing genetic reprogramming via engineered aminoacyl-tRNA synthetases. Both serve the goal of enhancing pharmacokinetic parameters either by offering functionalized groups for cyclisation or incorporation of elements that favor cell permeability (e.g., N-methylation) or protease resistance.

Although, orthogonal aminoacyl-tRNAs and ribosomes have been engineered within cells (Rackham and Chin, 2005), a favored method of introducing non-natural amino acids is to use cell-free *in vitro* transcription translation systems which offer greater flexibility and library capacity. Szostak's group studied the ability of wildtype aminoacyl-tRNA synthetases to mischarge tRNAs *in situ* with non-natural amino acids or the use of reconstituted cell-free transcription translation mixtures (Hartman et al., 2007; Schlippe et al., 2012). In total, over 50 substrates were found to be compatible with the *in vitro* expression system leading to ribosomal synthesis of a 15-mer peptide containing 13 non-natural amino acids. Although, most structures of these substrates were close analogs to their cognate amino acids, some included interesting functional groups as halo-aryl, alkene, azide and tert-butyl groups (Figure 2B). Although successful incorporation of N-methylated amino acids was possible, the yields obtained were low. This was attributed to the inefficiency of wildtype aminoacyl-tRNA synthetases to mischarge tRNAs rather than the ribosomal incorporation of such mischarged tRNAs into the nascent peptide chain. It was feasible to overcome this limitation by supplementation of the *in vitro* expression system with purified pre-charged tRNAs that had previously been methylated by chemical means (Subtelny et al., 2008, 2011).

These advances were applied to mRNA display for the selection of cyclic peptides containing non-natural amino acids. In the following study, a RNA library encoded random 10-mer peptides where 12 of the 20 proteinogenic amino acids were replaced by non-natural amino acids. The random region was flanked by two constant cysteine residues which were used to cyclise the peptide via the activated aryl α,α′-Dibromo-m-xylene. Selection against thrombin yielded two peptides of low nanomolar affinity in which cyclisation and incorporation of non-natural amino acids was required for binding to thrombin. This work exemplified the combination of both incorporation of non-natural amino acids and postranslational modification, however yet doesn't address the advantages in respect of proteolytic stability or oral bioavailability by incorporation of the non-natural amino acids (Schlippe et al., 2012).

The added functionality which can be introduced by incorporation of non-natural amino acids can also be used to cyclise peptides directly. 4-selenalysine can be used as substrate for lysine in an *in vitro* expression system and treated with hydrogen peroxide for oxidative elimination of the seleno moiety. The resulting dehydroalanine can subsequently react with cysteine to form a lantipeptide via a stable thioether bond (Figure 2C; Seebeck et al., 2011). The approach was applied to select lantipeptides from an mRNA encoded peptide library against Sortase A. Several lantipeptides could be identified, albeit with low μM affinities and the stereochemistry of the thioether bond had to be confirmed by synthesizing potential isomers (Hofmann et al., 2012). Natural lantipeptides represent an interesting class of macrocyclic modulators produced by a range of Gram-positive bacteria and are often characterized by antibacterial activities (Knerr and van der Donk, 2012). In future, therapeutic peptides might also be cyclized by the incorporation of dithiol amino acids which reduce the complexity of synthesizing disulphide rich sequences and have been demonstrated to significantly improve the activity of parental peptides that possess –CC- motifs. However, this has yet to be translated to biological systems (Chen et al., 2014).

An alternative to mischarge tRNAs with native aminoacyl tRNA synthetases enzymes has been developed based on flexizymes, a group of ribozymes that were engineered to aminoacylate tRNAs. These flexizymes were developed to have a low specificity toward the discriminatory nucleotides of a particular tRNA and, therefore, accept a broad spectrum of tRNA's as substrates. Three main flexizymes have been produced to recognize different leaving groups on activated amino acids leading to improved charging of the tRNAs with the non-natural amino acids based on the properties of the side chain. Orthogonal tRNAs, not recognized by wild type amino acyl tRNA synthesases, are used in place of native tRNAs and are accepted by the ribosome as substrates. Therefore, the problematic incorporation of competing proteinogenic amino acids during translation can be avoided. The generated non-natural amino acid charged tRNAs can then be added to an optimized *in vitro* expression system to fill codons within the genetic code that have been left vacant by withdrawing specific amino acids and their cognate aminoacyl-tRNA synthetases, therefore “reprogramming” the codon to the new components (Figures 2B,D; Ohta et al., 2007; Ohuchi et al., 2007; Xiao et al., 2008; Goto et al., 2011; Reid et al., 2012). This has recently been reviewed elsewhere (Passioura et al., 2014).

## Display independent approaches to enhance bioavailability

So far this report has focused on molecular display technologies which incorporate non-natural amino acids or post-translational modifications prior to selection so that compounds can be selected for affinity whilst incorporating elements to improve biophysical and biological properties. In theory, the bioavailability of a compound can be addressed following the isolation of a binder, however, in practice this has proven to be difficult. Conformational constrains or modifications that improve bioavailability often negatively affect the affinity with the exception of peptides in stable secondary structures. In particular the modification of α-helices with hydrocarbon staples has been shown to benefit both proteolytic stability and cell permeability. Although this approach is not always successful, attrition rates seem to be low if detailed structural data is available (Cromm et al., 2015). Similar methods have also been applied to β-turn structures (Obrecht et al., 2012).

A further variation presents the use of stable mini-scaffolds that are known to have good bioavailability but are still amendable to chemical synthesis. For example, cyclotides are small head-to-tail cyclised disulfide rich peptides, which have the potential to reach intracellular targets (Greenwood et al., 2007). These scaffolds can be used as hosts for grafted active sequences (Gould et al., 2011). Recently, recombinant expression of cyclotides in yeast by a split-intein approach coupled to a phenotypic screen has been developed thereby opening the doors to screen genetically encoded cyclotide-based libraries in eukaryotic cells (Jagadish et al., 2015).

## Future perspectives

Methods to modify natural peptides and those discovered *de novo* by man using directed evolution have generated peptidic ligands with improved properties, such as high affinity, excellent target specificity and good plasma stability; additional modifications can also provide pharmacological features to extend *in vivo* half-life, if necessary. Future developments may ultimately produce peptides that can be delivered orally, transverse the blood brain barrier or access intracellular targets. The selectivity of peptides, their rapid clearance by kidney filtration and capacity for chemical modification are particularly beneficial for molecular imaging applications and, potentially, for delivering toxic payloads as peptide drug conjugates. In terms of target interactions, peptides can occupy hydrophobic pockets favored by small molecules and therefore provide an attractive means for the discovery of druggable sites on proteins by acting as probes or templates for pharmaceutical drugs. These applications will see peptides becoming useful tools for target identification, validation in addition to their use as therapeutics.

### Conflict of interest statement

The author declares that the research was conducted in the absence of any commercial or financial relationships that could be construed as a potential conflict of interest. The authors have worked for Isogenica Ltd., a company which owns patents for CIS display.
